# Gut Microbiota and Antidiabetic Drugs: Perspectives of Personalized Treatment in Type 2 Diabetes Mellitus

**DOI:** 10.3389/fcimb.2022.853771

**Published:** 2022-05-31

**Authors:** Wenhui Liu, Zhiying Luo, Jiecan Zhou, Bao Sun

**Affiliations:** ^1^ Department of Pharmacy, The Second Xiangya Hospital, Central South University, Changsha, China; ^2^ Institute of Clinical Pharmacy, Central South University, Changsha, China; ^3^ Institute of Clinical Medicine, The First Affiliated Hospital, University of South China, Hengyang, China

**Keywords:** gut microbiota, antidiabetic drugs, type 2 diabetes mellitus, efficacy and safety, personalized therapeutic targets

## Abstract

Alterations in the composition and function of the gut microbiota have been reported in patients with type 2 diabetes mellitus (T2DM). Emerging studies show that prescribed antidiabetic drugs distort the gut microbiota signature associated with T2DM. Even more importantly, accumulated evidence provides support for the notion that gut microbiota, in turn, mediates the efficacy and safety of antidiabetic drugs. In this review, we highlight the current state-of-the-art knowledge on the crosstalk and interactions between gut microbiota and antidiabetic drugs, including metformin, α-glucosidase inhibitors, glucagon-like peptide-1 receptor agonists, dipeptidyl peptidase-4 inhibitors, sodium-glucose cotransporter 2 inhibitors, traditional Chinese medicines and other antidiabetic drugs, as well as address corresponding microbial-based therapeutics, aiming to provide novel preventative strategies and personalized therapeutic targets in T2DM.

## Introduction

Type 2 diabetes mellitus (T2DM) is a highly prevalent metabolic disorder characterized by elevated blood glucose levels, primarily caused by insulin secretion disturbance, insulin resistance, or both (Cersosimo et al., 2000). In recent years, gut microbiota, which refers to a complicated assembly of trillions of microbes, is reported to be involved in the pathogenesis and treatment responses of T2DM (Bouter et al., 2017; Koropatkin and Martens, 2017; Vázquez-Baeza et al., 2018). Additionally, emerging evidence has indicated that the gut microbiota affects the pharmacology of antidiabetic drugs, and drug-induced metabolites transform the structure of gut microbiota in turn (Gu et al., 2017; Koropatkin and Martens, 2017).

Gut microbiota is predominated by bacterial phyla *Firmicutes* and *Bacteroidetes*, followed by other phyla such as *Actinobacteria*, *Proteobacteria* and *Verrucomicrobia* (Woting and Blaut, 2016). With the growing recognition of gut microbiome as the second human genome, pharmacomicrobiomics has been introduced as the expansion of pharmacogenomics, which facilitates the investigation of the interaction between microbiome variation and drugs response (Doestzada et al., 2018). On the one hand, various studies have shown that antidiabetic drugs can affect the composition and function of gut microbiota (Forslund et al., 2015; Wu et al., 2017). On the other hand, the gut microbiota can influence an individual’s response to a specific drug by altering the drug’s bioactivity, bioavailability or toxicity (Koppel et al., 2017). A recent study showed that two-thirds of 271 tested drugs were subject to gut microbiota metabolism (Zimmermann et al., 2019), in which microbial enzymes transformed them into inactive or even toxic drug metabolites (Spanogiannopoulos et al., 2016). Despite the fact that interaction between gut microbiota and antidiabetic agents is increasingly being understood, the role of gut microbiota in the drug efficacy and safety is not fully clarified.

In the present review, we clarify the interaction between gut microbiota and antidiabetic agents, such as metformin, α-glucosidase inhibitors, glucagon-like peptide-1 (GLP-1) receptor agonists, dipeptidyl peptidase-4 (DPP4) inhibitors, sodium-glucose cotransporter 2 (SGLT2) inhibitors, traditional Chinese medicines (TCMs) and other antidiabetic drugs, as well as address the therapeutics based on gut microbiota, aiming to develop personalized treatments and potential individualized preventative and therapeutic strategies.

## Interaction Between Gut Microbiota and Antidiabetic Drugs

**Figure 1:**
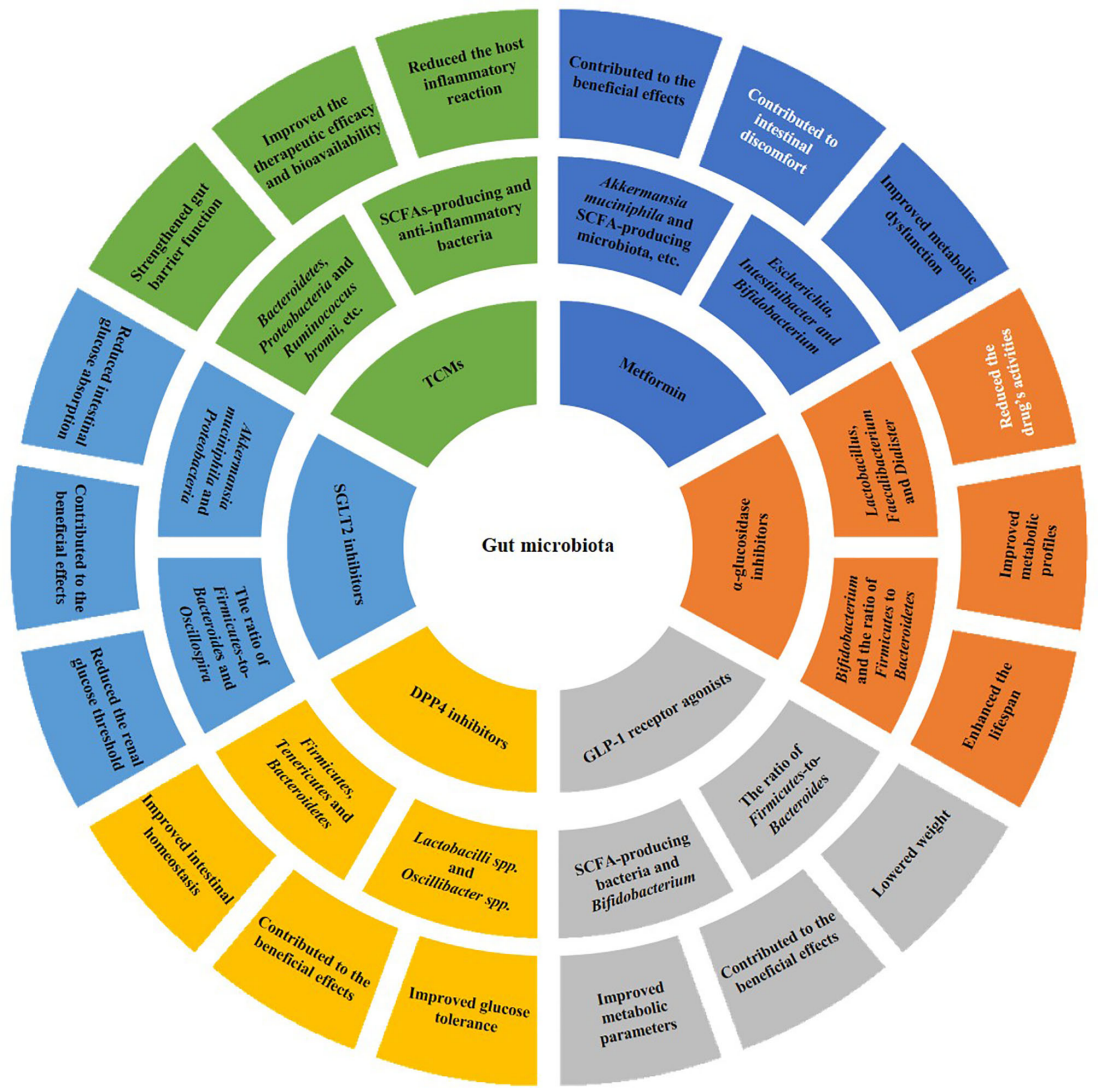
Bidirectional interaction between gut microbiota and antidiabetic drugs. On the one hand, antidiabetic drugs, including metformin, α-glucosidase inhibitors, GLP-1 receptor agonists, DPP4 inhibitors, SGLT2 inhibitors and TCMs, can affect the composition of gut microbiota (the second outermost circle). On the other hand, the gut microbiota can reduce the inflammatory reaction and alter the drug’s bioactivity, bioavailability or toxicity, thus influencing drug’s efficacy and safety, as well as improving metabolic dysfunction (the outermost circle). In the outermost ring, white fonts represent negative impacts of the antidiabetic drugs, while black fonts represent positive impacts. SCFA, short-chain fatty acid; GLP-1, glucagon-like peptide-1; DPP4, dipeptidyl peptidase-4; SGLT2, sodium-glucose cotransporter 2; TCMs, traditional Chinese medicines.

Lines of evidence have suggested that gut microbiota can not only be influenced by antidiabetic drugs (**Table 1**), but also in turn affect an individual’s response to those drugs. Furthermore, the interaction between gut microbiota and antidiabetic drugs is complex and bidirectional (**Figure 1**).

**Table 1:** Effect of antidiabetic drugs on gut microbiota in T2DM.

Antidiabetic drugs	Changes in gut microbiota	Mechanisms	References
Metformin	Increased *Escherichia* and lowered *Intestinibacter* abundance	NA	Forslund et al., 2015
	Increased *Escherichia* and *Bifidobacterium*, as well as lowered *Intestinibacter* abundance	Affected pathways and regulated genes encoding metalloproteins or metal transporters	Wu et al., 2017
	Enriched the abundance of *Akkermansia muciniphila* and SCFA-producing microbiota	NA	de la Cuesta-Zuluaga et al., 2017
	Increased *Enterobacteriales* and *Akkermansia muciniphila*	NA	Cao et al., 2020
	Increased *E. coli* and *R. torques* and decreased *I. bartlettii* and *R. intestinalis* at 6 and 12 months	NA	Mueller et al., 2021
α-glucosidase inhibitors	Increased abundance of *Bifidobacterium longum* and decreased concentration of lipopolysaccharides	Decreased levels of related cytokines and alleviated the inflammatory status	Su et al., 2015
	Increased *Lactobacillus*, *Faecalibacterium*, and *Dialister* and decreased *Butyricicoccus, Phascolarctobacterium* and *Ruminococcus*	NA	Zhang X. et al., 2017
	Contributed to the plentitude of *Bifidobacterium* and *Lactobacillus*	Promoted amino acid pathways	Zhang F. et al., 2019
	Decreased the ratio of *Firmicutes* to *Bacteroidetes*	Downregulated expression levels of CYP8B1 and HNF4α genes and upregulated PGC1α	Do et al., 2016
GLP-1 receptor agonists	Increased the ratio of *Firmicutes*-to-*Bacteroides*	NA	Wang et al., 2016; Zhao et al., 2018
	Elevated SCFA-producing bacteria and *Bifidobacterium*	NA	Zhang et al., 2018
	Increased the frequency of the *Bacteroidetes* to *Firmicutes* phyla ratio	Reduced the frequency of Th1 lymphocytes, as well as increased TReg and ILC1 and 3 cells	Charpentier et al., 2021
DPP4 inhibitors	Increased the abundance of *Bacteroidetes*	NA	Liao et al., 2019
	Increased *Firmicutes* and *Tenericutes*, as well as decreased *Bacteroidetes*	NA	Yan et al., 2016; Zhang Q. et al., 2017
	Increased *Lactobacilli* spp. and propionate production along with decreased *Oscillibacter* spp.	Restored the expression of AMPs and the depth of the crypts in the ileum	Olivares et al., 2018a
SGLT2 inhibitors	Decreased *Firmicutes*-to-*Bacteroidetes* ratio and *Oscillospira*, as well as increased *Akkermansia muciniphila*	NA	Lee et al., 2018
	Increased the relative abundance of *Proteobacteria* and did not influence the abundance of the *Firmicutes*-to-*Bacteroidetes* ratio	NA	Yang et al., 2020
	Almost did not change	NA	Du et al., 2018; van Bommel et al., 2020
TCMs	Increased the relative abundance of *Bacteroidetes* and decreased *Proteobacteria*	NA	Yao et al., 2020; Xu et al., 2021
	Inhibited *Ruminococcus bromii*	Attenuated DCA transformation	Zhang Y. et al., 2020
	Enriched butyrate-producing bacteria	Induced ileal gene expression and relieved systemic and local inflammation	Xu et al., 2020
	Increased SCFAs-producing and anti-inflammatory bacteria	NA	Chen et al., 2018; Wei et al., 2018
	Enriched *Akkermansia muciniphila* and SCFAs level	Strengthened gut barrier function and reduced the host inflammatory reaction	Cao et al., 2019; Su et al., 2020
	Up-regulated *Firmicutes* and *Lactobacillus*	Up-regulated PBA-FXR-GLP-1 pathway	Chen et al., 2021
Insulin	Increased the abundance of *Fusobacterium*	Up-regulated the genes involved in triglyceride and arachidonic acid metabolism	Zhang F. et al., 2019

T2DM, type 2 diabetes mellitus; NA, not available; SCFA, short-chain fatty acid; AMP, antimicrobial peptide; GLP-1, glucagon-like peptide-1; DPP4, dipeptidyl peptidase-4; SGLT2, sodium-glucose cotransporter 2; TCMs, traditional Chinese medicines; DCA, deoxycholic acid; PBA, primary bile acid; FXR, farnesoid X receptor.

### Antidiabetic Drugs Influencing the Gut Microbiota

Metformin is the most commonly used glucose-lowering drug for the treatment of T2DM, especially T2DM associated with obesity. Previous studies indicated that intravenous administration of metformin did not lower glucose in contrast to oral metformin (Bonora et al., 1984), and the bioactivity of metformin originated in the intestine (Bailey et al., 2008). There is evidence to suggest that metformin changes microbiota composition in not only T2DM patients (Forslund et al., 2015; Wu et al., 2017; Nakajima et al., 2020), but also in healthy people (Bryrup et al., 2019; Ejtahed et al., 2019). Metagenomic analysis of microbiota suggested that metformin influenced antidiabetic effect through short-chain fatty acids (SCFAs) production, as well as potential microbial genes and pathways (Lee and Ko, 2014; Forslund et al., 2015; de la Cuesta-Zuluaga et al., 2017; Wu et al., 2017; Bauer et al., 2018). In addition, increase in the production of SCFAs, especially butyrate and propionate, activated intestinal gluconeogenesis, which improved glycemic control and reduced hepatic glucose production, appetite and body weight (Ejtahed et al., 2016). To elucidate the mechanism by which gut microbiota mediated the antidiabetic effects of metformin, a further study investigated metformin-microbiota interactions and showed that metformin affected pathways with biological functions in species from mucin-degradation bacteria and SCFA production, and related genes in these species encoded metalloproteins or metal transporters (Wu et al., 2017). A systematic review stressed that the changes of gut microbiota were associated with metformin, and T2DM patients receiving metformin showed increases in *Enterobacteriales* and *Akkermansia muciniphila*, a mucin-degrading bacteria that has been shown to reverse metabolic disorders (Cao et al., 2020). On the other hand, a randomized trial reported that metformin shifted long-term gut microbiota composition, increasing *E. coli* and *R. torques* and decreasing *I. bartlettii* and *R. intestinalis* at 6th and 12nd month in overweight and obese cancer survivors, respectively (Mueller et al., 2021). Furthermore, in healthy subjects without changes in glycemic control, metformin led to an increased abundance of *Escherichia/Shigella* spp. and *Bilophila wadsworthia*, as well as a reduced abundance of *Clostridium* spp. and *Intestinibacter* spp. (Bryrup et al., 2019). These results suggest that the changes in microbiota were caused by metformin itself, rather than simply reflecting improved glycemic control.

To figure out the association between diabetes and gut microbiota modified by metformin, Cuesta-Zuluaga et al. performed a retrospective study and found that patients with diabetes taking metformin had higher relative abundance of mucin-degradation *Akkermansia muciniphila* and several sorts of SCFA-producing microbiota compared with participants without diabetes (de la Cuesta-Zuluaga et al., 2017). Conversely, for diabetic patients not taking metformin, relative abundance was higher in *Clostridiaceae* 02d06 and lower in *Enterococcus casseliflavus* (de la Cuesta-Zuluaga et al., 2017).

α-glucosidase inhibitors, including acarbose, voglibose and miglitol, are the first-line drugs in noninsulin-dependent T2DM characterized by their high efficacy in postponing the digestion of carbohydrates and reducing postprandial hyperglycemia (Montandon and Jornayvaz, 2017), those medications inhibit carbohydrate hydrolysis by binding to human intestinal maltase-glucoamylase (MGAM) and sucrase-isomaltase (SI), and consequently delay and reduce the absorption of glucose. Furthermore, there is growing evidence that α-glucosidase inhibitors impact microbiota composition. For instance, T2DM patients treated with acarbose showed increased abundance of *Bifidobacterium longum* and decreased concentration of lipopolysaccharides (Su et al., 2015). Another clinical trial suggested that *Butyricicoccus, Phascolarctobacterium*, and *Ruminococcus* decreased while *Lactobacillus*, *Faecalibacterium*, and *Dialister* increased in patients with prediabetes after acarbose treatment (Zhang X. et al., 2017). Interestingly, Smith et al. observed that there were notable changes in microbial communities and the concentrations of SCFAs in the mice treated with acarbose compared with those of control mice, and microbial communities and fecal SCFAs increased the lifespan of the mice treated with acarbose (Smith et al., 2019). Recently, a study from Chinese population provided that α-glucosidase inhibitors contributed to the plentitude of *Bifidobacterium* and *Lactobacillus*, as well as promoted several amino acid pathways (Zhang F. et al., 2019). Also, 12-week voglibose administration decreased the ratio of *Firmicutes* to *Bacteroidetes* and improved metabolic profiles including those of blood glucose and lipid metabolism (Do et al., 2016). Therefore, it has been suggested that α-glucosidase inhibitors may have beneficial effects on glycemic control partly through gut microbiota in T2DM.

GLP-1 is an incretin hormone secreted by intestinal endocrine cells (L cells) in response to food ingestion (Drucker and Nauck, 2006). It can enhance glucose-induced insulin from pancreatic β-cells and suppress glucagon secretion; in addition, it also contributes to the inhibition of appetite and gastric emptying (Drucker and Nauck, 2006; Baggio and Drucker, 2014). Numbers of studies have shown that gut microbiota modulates satiety and glucose homoeostasis by inducing the secretion of GLP-1 in mice (Tolhurst et al., 2012; Kimura et al., 2013; Vettorazzi et al., 2016; Aoki et al., 2017). Meanwhile, GLP-1 receptor agonists, a new class of antidiabetic drugs, were also reported to affect the intestinal environment and, indeed, changes in the gut microbiota had been linked to GLP-1 receptor agonists (Wang et al., 2016; Zhang et al., 2018; Zhao et al., 2018; Charpentier et al., 2021; Shang et al., 2021). Generally, the *Firmicutes* to *Bacteroidetes* ratio is regarded to be of significant relevance in human gut microbiota composition. Wang et al. demonstrated that liraglutide could modulate the gut microbiota to a more lean-related composition in diabetic mice with normal weight, and they also found a higher *Firmicutes*-to-*Bacteroides* ratio after liraglutide treatment (Wang et al., 2016). Inconsistently, another study argued that liraglutide increased the *Bacteroides*-to-*Firmicutes* ratio to lower weight significantly regardless of the glycemic status in both simple obese and diabetic obese subjects (Zhao et al., 2018). This discrepancy may be attributed to the different level of hyperglycemia and model systems used. By constructing diabetic animal model, researches showed that administration of GLP-1 receptor agonists profoundly changed the composition of gut microbiota in diabetic male rats (Yuan et al., 2018; Zhang et al., 2018). In particular, several SCFAs-producing bacteria, including *Bacteroides*, *Lachnospiraceae*, and probiotic bacteria, including *Bifidobacterium*, were selectively enhanced in liraglutide-treated diabetic male rats (Zhang et al., 2018). In parallel, liraglutide increased the *Bacteroidetes-*to-*Firmicutes* ratio by reducing the Th1 cell frequency and enhancing certain immune cells, such as regulatory T (Treg) cell, innate lymphoid cell 1 (ILC1) and ILC3, which was linked to the nitrogen or the purine metabolism pathways, thus improving glucose-induced insulin secretion (Charpentier et al., 2021). Crucially, GLP-1 receptor agonists could at least partially restore the balance of gut microbiota (Yuan et al., 2018).

DPP4 inhibitors have been proposed to lower blood glucose primarily through inhibiting the degradation of GLP-1 and are recommended as a first-line hypoglycemic treatment in T2DM by the American Association of Clinical Endocrinologists (Drucker and Nauck, 2006; Handelsman et al., 2015). A previous study proposed the DPP-4-like activity of the gut microbiota as a target of DPP-4 inhibition, which could open new therapeutics uses of DPP4 inhibitors to regulate gut microbiota dysbiosis (Olivares et al., 2018b). Liao et al. demonstrated that DDP4 inhibitors improved glucose metabolism by increasing the abundance of *Bacteroidetes*, and substantially reversing the changes in the gut microbiota induced by high fat diet (HFD) (Liao et al., 2019). An investigation into the effect of sitagliptin on gut microbiota indicated that the phyla *Bacteroidetes* decreased, while *Firmicutes* and *Tenericutes* increased; in addition, sitagliptin partially corrected the dysbiosis of microbiota and altered the population of SCFA-producing bacteria in HFD-fed rats with T2DM (Yan et al., 2016). Similarly, another experiment showed that vildagliptin treatment was associated with increased *Bacteroidetes* and decreased *Firmicutes* along with decreased *Firmicutes/Bacteroidetes* ratio in the diabetic rats (Zhang Q. et al., 2017). In parallel, vildagliptin was proposed to exert beneficial effects through the modulation of gut microbiota, and was linked with increased *Lactobacilli* spp. and propionate production along with decreased *Oscillibacter* spp. (Olivares et al., 2018a). To explain these changes, Olivares and his colleagues performed experiments and found that vildagliptin reduced Toll-like receptor (TLR) ligands in caecal content, as well as restored the expression of antimicrobial peptides (AMPs) and the depth of the crypts in the ileum (Olivares et al., 2018a). Furthermore, they also explored that vildagliptin indirectly reduced gene expression of proinflammatory cytokines in liver. These findings demonstrate an important effect of DPP4 inhibitors on the gut microbiota, revealing a potential strategy for improving glucose homeostasis.

SGLT2 inhibitors, a novel class of anti-diabetic substances, are used to achieve the glucose-lowering effect by increasing urinary glucose excretion (Tahrani et al., 2013). After 8 weeks of treatment with dapagliflozin, diabetic mice displayed lower arterial stiffness and blood glucose level, and even more importantly, decreased *Firmicutes*-to-*Bacteroidetes* ratio and *Oscillospira*, as well as increased *Akkermansia muciniphila* (Lee et al., 2018). Notably, another study demonstrated that dapagliflozin and metformin have similar glucose-lowering effect, but they differentially affected the composition of fecal microbiota in type 2 diabetic rats (Yang et al., 2020). The dapagliflozin group mainly increased the relative abundance of *Proteobacteria* (especially *Desulfovibrionaceae*) and did not influence the *Firmicutes*-to-*Bacteroidetes* ratio. Conversely, several studies considered that SGLT2 inhibitors had almost no effect on gut bacteria (Du et al., 2018; van Bommel et al., 2020). Nevertheless, it was essential to emphasize that all the study participants had been treated with metformin, which could have overshadowed the potential effects of dapagliflozin on the gut microbiota (van Bommel et al., 2020). In short, further research is needed to figure out the influence of SGLT2 inhibitors on gut microbiota.

TCMs, generally also known as botanical medicine or phytomedicine, have been shown to effectively reduce blood glucose for many years (Lian et al., 2015; Hu et al., 2016). Although TCMs have significant effects on the treatment of T2DM, the mechanisms underlying the therapeutics effects remain elusive. In recent decades, accumulating evidence confirmed that TCMs could improve T2DM by modulation of gut microbiota (Xu et al., 2015; Nie et al., 2019; Zhang B. et al., 2019; Zheng et al., 2020a; Zheng et al., 2020b). Yao et al. observed that Berberine reduced the blood glucose levels and improved glucose tolerance and serum lipid parameters in type 2 diabetic rats (Yao et al., 2020). Further analysis found that the relative abundance was increased for *Bacteroidetes* and decreased for *Proteobacteria* and *Verrucomicrobia* after Berberine treatment (Yao et al., 2020). Likewise, after 30 days of administration, *Bereris kansuensis* extract increased the abundance of phyla *Bacteroidetes* and *Akkermansia*, while reduced the abundance of *Proteobacteria* and several harmful bacteria (e.g., *Enterococcus* and *Fusobacterium*), which was related to its antidiabetic effect in T2DM rats (Xu et al., 2021). To investigate the potential microbial-related mechanism underlying the hypoglycemic effect of Berberine, Zhang et al. found that the inhibition of deoxycholic acid (DCA) biotransformation by *Ruminococcus bromii* might be involved in the hypoglycemic effect of Berberine (Zhang Y. et al., 2020). Moreover, a recent study demonstrated that the glucose-lowering effect of Gegen Qinlian Decoction could be attributed to Berberine, and both of them significantly modulated the overall gut microbiota structure and enriched butyrate-producing bacteria, including *Faecalibacterium* and *Roseburia* (Xu et al., 2020). Additionally, two TCM prescriptions, Xiexin Tang and Huang-Lian-Jie-Du-Decoction were reported to increase SCFAs-producing and anti-inflammatory bacteria (e.g., *Parabacteroides*, *Blautia*, *Akkermansia*, and *Adlercreutzia*) in T2DM rats (Chen et al., 2018; Wei et al., 2018), providing novel insights into the mechanism and clinical treatment for T2DM from the perspective of gut microbiota. Another two TCMs, JinQi Jiangtang tablets and Andrographolide ameliorated glucose intolerance and insulin resistance in T2DM mice by enriching microbial species of *Akkermansia muciniphila* and increasing SCFAs level (Cao et al., 2019; Su et al., 2020). The mechanism was related to regulating the gut barrier integrity and reducing the host inflammation. Consistent with the abovementioned results, Chen et al. found that Ge-Gen-Jiao-Tai-Wan formula could reduce blood glucose levels and improve glucose tolerance by regulating the composition of the gut microbiota (Chen et al., 2021). Correspondingly, Ge-Gen-Jiao-Tai-Wan formula up-regulated the beneficial phylum *Firmicutes* and bile-acid-related genus *Lactobacillus*, promoting the production of primary bile acids (PBAs) and activating the PBA- farnesoid X receptor (FXR)-GLP-1 pathway (Chen et al., 2021).

In addition to the above-mentioned antidiabetic drugs, the influence of sulfonylurea, peroxisome proliferative activated receptor (PPARG) agonists and insulin on microbiota composition and the consequent metabolic benefits has also been emphasized (Huo et al., 2015; Zhang F. et al., 2019; Madsen et al., 2021). Insulin increased the abundance of *Fusobacterium*, which up-regulated the genes involved in triglyceride and arachidonic acid metabolism (Zhang F. et al., 2019). Previous studies reported that hippurate was a component of urine and mainly generated from the breakdown of plant phenols and aromatic amino acids by gut microbiota (Williams et al., 2002; Mulder et al., 2005). Metabonomic analysis investigated that levels of those aromatic amino acids (phenylalanine and tryptophan) were decreased, and hippurate was increased in the urine of T2DM patients after the treatment of sulfonylurea, which might be mediated *via* gut microbiota (Huo et al., 2015). Recently, Madsen et al. revealed that rosiglitazone, a PPARG agonist, improved glucose homeostasis without influencing local gut microbiome in diabetic db/db mice by using full-length bacterial 16S rRNA sequencing (Madsen et al., 2021).

### The Impact of Gut Microbiota on Antidiabetic Drug’s Efficacy and Safety

Although the changes in gut microbiota caused by antidiabetic drugs were not simply reflecting improved glycemic control, the antidiabetic effect and safety of antidiabetic agents depended partly on certain groups of gut microbiota (**Table 2**). Wu et al. transferred the fecal samples from metformin-treated donors (treated with metformin for 4 months) to germ-free mice and indicated that glucose tolerance was improved mainly through increasing the production of SCFAs or altering plasma bile acid composition, suggesting that increased growth of SCFA-producing bacterial species could potentially contribute to the antidiabetic effect of metformin (Wu et al., 2017). Another study revealed that the level of bile acid glycoursodeoxycholic acid (GUDCA) was increased and *Bacteroides fragilis* was decreased in newly diagnosed T2DM treated with metformin for 3 days (Sun et al., 2018). Further experiments confirmed that *B. fragilis*–GUDCA–intestinal FXR axis mediated the glucose-lowering effect of metformin.

**Table 2:** Impact of gut microbiota on antidiabetic drug’s efficacy and safety in T2DM.

Related changes in gut microbiota	Antidiabetic drugs	Impact of efficacy or safety	References
Increased abundance of *Escherichia* species	Metformin	Contributed to intestinal discomfort	Forslund et al., 2015
Increased SCFAs or bile acid composition	Metformin	Contributed to the beneficial effects	Wu et al., 2017
Decreased *Bacteroides fragilis* and increased the bile acid GUDCA	Metformin	Improved metabolic dysfunction	Sun et al., 2018
Enriched *Blaubia obeum*	α-glucosidase inhibitors	Reduced their efficacy	Tan et al., 2018
Increased abundance of the phylum *Firmicutes* and *Bacteroidetes*	DPP4 inhibitors	Improved glucose tolerance and contributed to hypoglycemic effect	Liao et al., 2019
Increased NR-producing bacteria	TCMs	Improved the therapeutic efficacy and bioavailability	Wang et al., 2017

T2DM, type 2 diabetes mellitus; SCFA, short-chain fatty acid; GUDCA, glycoursodeoxycholic acid; DPP4, dipeptidyl peptidase-4; TCMs, traditional Chinese medicines; NR, nitroreductase.

α-glucosidase inhibitors, which were not absorbed in the small intestine or not metabolized before excretion, created a chance for unintended cross-interaction with gut microbiota. Previous studies identified that the sequence and structural active sites of human intestinal α-glucosidases (MGAM and SI) and microbial α-glucosidases (from *Blaubia obeum*) were highly homologous, and microbial α-glucosidases could process dietary carbohydrates as well as be inhibited by α-glucosidase inhibitors with comparable strengths (Kuriyama et al., 2008; Natori et al., 2011). Thus, the location and any changes of these active sites might affect the access and specificity of these α-glucosidases to α-glucosidase inhibitors (Tan et al., 2018), mediating their therapeutic effect.

GLP-1 resistance has been reported to seriously impair the effect of GLP-1 receptor agonists (Knop et al., 2012). Grasset et al. identified a specific set of ileum bacteria impairing the GLP-1-activated gut brain axis for the control of insulin secretion and gastric emptying, hence inducing GLP-1 resistance (Grasset et al., 2017). Intriguingly, fecal samples from DDP4 inhibitors-treated T2DM patients transferred to HFD-fed mice improved the glucose intolerance of the recipients, suggesting that the altered gut microbiota contributed to hypoglycemic effects of DDP4 inhibitors even in the absence of additional treatments (Liao et al., 2019). In addition, the gut microbiota might improve the therapeutic efficacy and bioavailability of TCMs by affecting their transformation and absorption (Wang et al., 2017).

In addition to the impact on drug efficacy, gut microbiota can also contribute to the side effects of antidiabetic drugs. It is well known that gastrointestinal side effects are reported in up to one-third of patients taking metformin, and these side effects can be attributed to the identified metabolism genes (mainly derived from an increase of *E. coli* species) and the increase of virulence factors (Forslund et al., 2015). Because of the high homology of the active sites of human α-glucosidases and gut bacterial α-glucosidases, one proposed theory was that α-glucosidase inhibitors could affect the bacterial α-glucosidases in human gut and exert beneficial effects or create adverse gastrointestinal symptoms (Tan et al., 2018).

## New Insights for Developing Personalized Treatments

**Figure 2:**
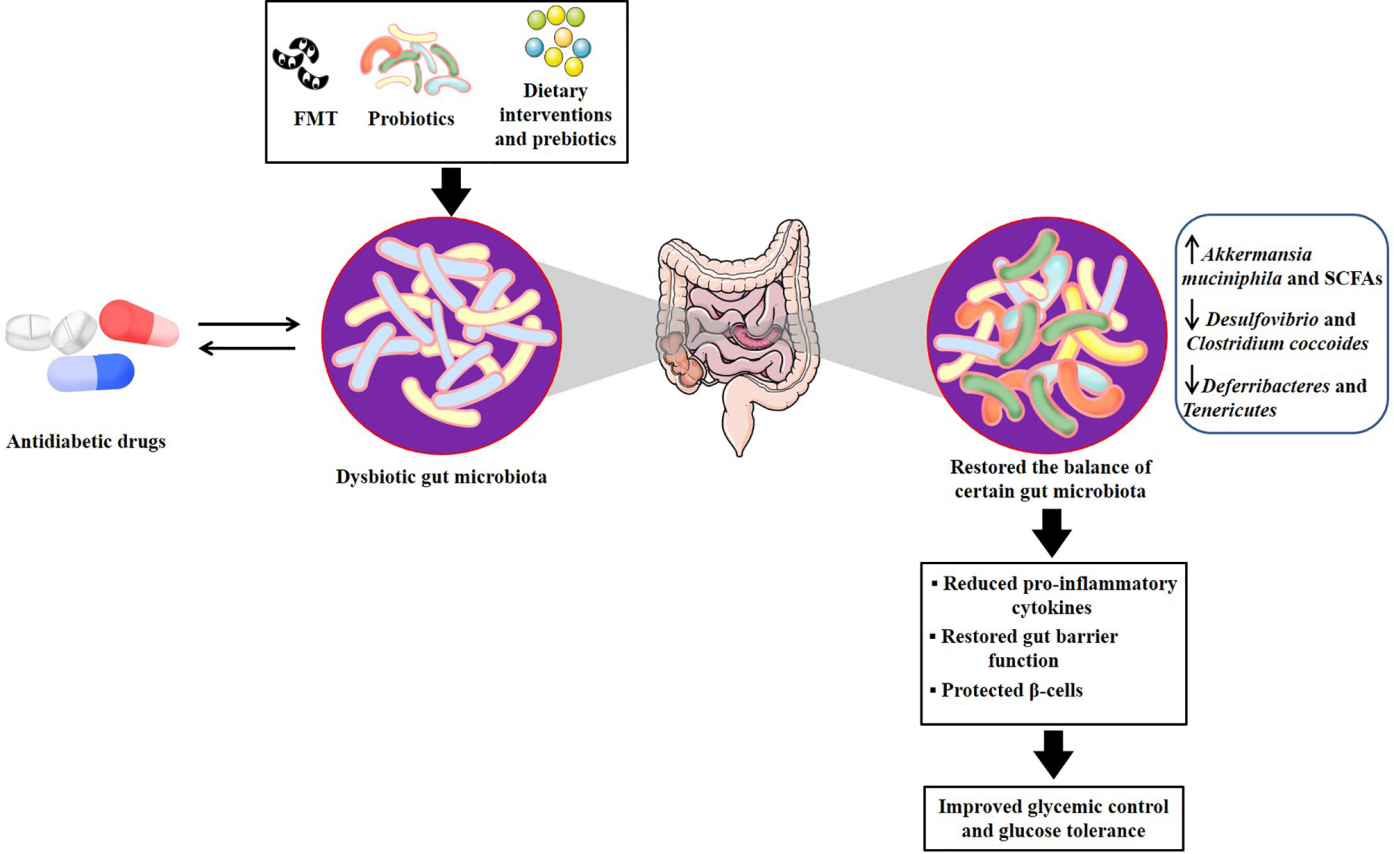
Potential mechanisms of microbial-based therapeutics for developing personalized treatments in T2DM. There are several relevant mechanisms through which antidiabetic drugs treat T2DM by regulating the gut microbiota. Microbial-based therapeutics, including FMT, probiotics and dietary interventions and prebiotics, could directly target gut microbiota or act as an adjunctive to antidiabetic drugs to restore the balance of several certain dysbiotic gut microbiota, which contributed to reducing pro-inflammatory cytokines, restoring gut barrier function, as well as protecting β cells, therefore improving glycemic control and glucose tolerance. FMT, fecal microbiota transplantation; T2DM, type 2 diabetes mellitus.

Given the interplay between gut microbiota and antidiabetic drugs, there is increasing awareness that altering microbiota can impact metabolic phenotype and provide a rational basis for targeting gut microbiota to develop personalized treatments in T2DM (Aron-Wisnewsky et al., 2019; Ghorbani et al., 2021; Huda et al., 2021). Several new insights including fecal microbiota transplantation (FMT), probiotics or prebiotics, and intermittent-fasting could contribute to the desired drug response and personalized medicine (**Table 3** and **Figure 2**).

**Table 3:** Potential microbial-based therapeutics for developing personalized treatments in T2DM.

Microbial-based therapeutics	Subjects	Results	References
FMT	Mice with diabetes	Increased the fecal levels of *Akkermansia muciniphila*, decreased *Desulfovibrio* and *Clostridium coccoides* levels and lowered fasting blood glucose concentrations	Zhang P.P. et al., 2020
	Germ-free mice	Increased SCFAs and bile acid composition, as well as improved glucose tolerance	Wu et al., 2017
	Metabolic syndrome patients	Increased fecal acetate or butyrate at 6 weeks	Kootte et al., 2017
Probiotics	(STZ+HFD)-induced T2DM mice	Increased the *Akkermansiaceae* family and SCFAs, as well as protected β-cells and alleviated hyperglycemia	Lee et al., 2021
	(STZ+HFD)-induced T2DM rats	Protected β-cells, stabilized glycemic levels and reduced inflammation	Hsieh et al., 2021
	T2DM patients	Increased the level of SCFAs	Toejing et al., 2021
	T2DM patients	Decreased fasting plasma glucose and insulin resistance	Palacios et al., 2020
Dietary interventions and prebiotics	T2DM patients	Improved lipid metabolism and glucose homeostasis	Mahboobi et al., 2018
	T2DM mice	Reduced the blood glucose level and oral glucose tolerance level, as well as increased the level of SCFAs and improved biochemical parameters	Yu et al., 2021
	T2DM patients	Increased concentrations of faecal SCFAs with six weeks supplementation of inulin-type fructans	Birkeland et al., 2020
	T2DM mice	Reduced abundance of *Deferribacteres* and *Tenericutes*, and suppressed inflammation	Li et al., 2019

FMT, fecal microbiota transplantation; STZ, streptozotocin; HFD, high fat diet; T2DM, type 2 diabetes mellitus; SCFA, short-chain fatty acid.

### FMT

FMT is a process of transferring stool from a healthy donor to another patient’s intestinal tract, which can not only reverse gut microbiota dysbiosis (He et al., 2015), but also rebuild the recipient’s intestinal microbial ecosystem (Groen and Nieuwdorp, 2017). Over the past few years, T2DM has been suggested responsive to FMT (Khoruts and Sadowsky, 2016), which also has attracted increased attention.

In the FMT-treated mice with diabetes, *Desulfovibrio* and *Clostridium coccoides* levels were significantly decreased, and the fecal levels of *Akkermansia muciniphila* were increased (Zhang P.P. et al., 2020). Moreover, *Akkermansia muciniphila* led to increased expression of HDAC3, which remarkably improved glycolipid metabolism. Likewise, glucose tolerance was improved by transfer of fecal samples from patients treated with metformin to germ-free mice (Wu et al., 2017). Metagenomics analysis indicated that metformin promoted functional shifts in gut microbiota of fecal samples, including lipopolysaccharide biosynthesis and SCFA metabolism. Notably, another study investigated the effects of lean donor-FMT versus self-FMT on patients with metabolic syndrome and found that insulin sensitivity was not changed at 18 weeks after self-FMT, but was significantly improved at 6 weeks after lean donor-FMT (Kootte et al., 2017). Moreover, a recent review considered that whether FMT was a future therapeutic option needed further evaluation (Aron-Wisnewsky et al., 2019). Collectively, FMT may be an interesting option to modify certain gut microbiota and a potential target for developing personalized treatments.

### Probiotics

Probiotics are live microorganisms, which have a beneficial effect on human health when administered in adequate amounts (Hill et al., 2014; Kesika et al., 2019). A number of studies revealed that multi-strain probiotic supplement, including *Lactobacillus plantarum* HAC01 and *Probioglu™*, lowered blood glucose and HbA1c levels, as well as improved glucose tolerance by protecting β-cells and restoring the gut microbiota and SCFAs in streptozotocin-induced diabetic rat models with HFD (Hsieh et al., 2021; Lee et al., 2021). Furthermore, a randomized clinical trial performed by Toejing et al. demonstrated that probiotic supplementation *L. paracasei* HII01 significantly decreased fasting blood glucose level by increasing beneficial bacteria and decreasing pathogenic bacteria, thus suggesting a potential role of this probiotic as an adjuvant treatment in T2DM (Toejing et al., 2021). Another randomized controlled pilot study showed that participants taking metformin in combination with probiotics had higher concentration of plasma butyrate and SCFA-producing bacteria after the 12-week intervention, lower fasting plasma glucose and weaker insulin resistance, which suggested that probiotic might act as an adjunctive to metformin and thus enhanced glucose management at the individual level (Palacios et al., 2020). Consistent with this result, a recent study showed that the probiotic supplementation improved the glycemic parameters in T2DM patients and thus could be recommended as a potential adjuvant treatment alongside medicine for T2DM therapy (Bock et al., 2021). Mechanistically, these probiotics exerted antidiabetic effect and ameliorated the symptom of T2DM, as well as restored gut barrier function *via* reducing pro-inflammatory cytokines and intestinal permeability, and activating antioxidant enzymes (Sharma et al., 2016; Kim et al., 2018; Wang et al., 2020).

### Dietary Interventions and Prebiotics

Dietary interventions and prebiotics are the ingredients that beneficially affect the host by selectively promoting the growth and the activity of certain bacterial species (Gibson and Roberfroid, 1995; Wu et al., 2011). Although gut microbiota played an important role in human by interacting with host diet, there was large inter-individual variation in the response to diet (Lampe et al., 2013), and studies displayed that the gut microbial composition could be used to identify those participants who would benefit from dietary interventions or prebiotics (Korpela et al., 2014; Salonen et al., 2014; Kovatcheva-Datchary et al., 2015). A meta-analysis of randomized controlled trials concluded that dietary interventions supplemented with either prebiotics or synbiotics resulted in improvements in glucose homeostasis in patients with T2DM (Mahboobi et al., 2018). Moreover, Yu et al. argued that different dietary supplements might exert synergistic protective effects against T2DM *via* reducing the blood glucose levels and effectively improving some beneficial bacterium (Yu et al., 2021).

Additionally, prebiotic inulin was conducive to alleviate T2DM by modulating gut microbiota (Li et al., 2019; Birkeland et al., 2020). Further analysis found that dietary inulin increased the relative abundance of *Cyanobacteria and Bacteroides*, as well as reduced the relative abundance of *Ruminiclostridium*, *Deferribacteres*, and *Tenericutes via* suppressing inflammation (Li et al., 2019). Noteworthily, a symbiotic mixture of prebiotics and probiotics supplementation could be more beneficial compared to prebiotic or probiotic alone (Morshedi et al., 2020). A recent randomized trial also demonstrated that administration of berberine with probiotics improved blood glucose levels compared to the group treated with berberine alone (Zhang Y. et al., 2020). Therefore, there will be a promising synergistic approach in the future involving both diet and prebiotics in the personalized prevention and treatment of T2DM. Currently, a prominent study integrated clinical and microbial data and devised machine learning algorithms for postprandial glycemic response (PPGR) prediction (Shilo et al., 2022), which implied that personally tailored treatments could be customized for individuals in the future.

## Conclusions and Future Perspectives

With the bidirectional interaction between gut microbiota and antidiabetic agents is increasingly being understood, targeting gut microbiota can contribute to increasing drug efficacy and safety, and thus enable a personalized medicine approach for the treatment and management of T2DM.

It is noteworthy that pharmacomicrobiomics play an essential role in combing personal microbiome and genetic profiles to better predict individual’s drug response and efficacy at the individual level. Mapping and modeling human microbiome drug metabolism with genome-scale and meta-omics analyses could improve our understanding of the roles of drugs and microbial communities in personalized medicine (Zimmermann et al., 2019; Javdan et al., 2020; Heinken et al., 2021). With the advance of multi-omics in gut microbiota research, microbiota-based personalized treatment is expected to be achieved by integration of multi-omics data with microbiome data in T2DM patients. Excitingly, thanks to current technologies, around 80% of gut microbes are readily available using bacterial culture (Lagier et al., 2016), which helps to mimic the intestinal environment and makes it possible to conduct individual-based drug testing on cultured bacteria, thus developing novel preventative strategies and personalized therapeutic targets.

Finally, applying the abovementioned novel approaches may contribute to a better understanding of the interactions between gut microbiota and antidiabetic drugs in T2DM, ultimately leading to future potential major advances in personalized medicine.

## Author Contributions

WL, ZL and JZ wrote the original draft. WL and ZL reviewed and edited the draft. BS revised and supervised overall project. All authors read and approved the final version of manuscript. All authors contributed to the article and approved the submitted version.

## Funding

This work was supported by grants from National Natural Science Foundation of China (No. 82104307, 82003872), Natural Science Foundation of Hunan Province (No. 2021JJ40865, 2021JJ40847, 2020JJ5513).

## Conflict of Interest

The authors declare that the research was conducted in the absence of any commercial or financial relationships that could be construed as a potential conflict of interest.

## Publisher’s Note

All claims expressed in this article are solely those of the authors and do not necessarily represent those of their affiliated organizations, or those of the publisher, the editors and the reviewers. Any product that may be evaluated in this article, or claim that may be made by its manufacturer, is not guaranteed or endorsed by the publisher.

## Abbreviations

T2DM, Type 2 diabetes mellitus; GLP-1, glucagon-like peptide-1; DPP4, dipeptidyl peptidase-4; SGLT2, sodium-glucose cotransporter 2; TCMs, traditional Chinese medicines; SCFAs, short-chain fatty acids; PPARG, peroxisome proliferative activated receptor; MGAM, maltase-glucoamylase, SI, sucrase-isomaltase; Treg, regulatory T; ILC1, innate lymphoid cell 1; HFD, high fat diet; TLR, Toll-like receptor; AMPs, antimicrobial peptides; DCA, deoxycholic acid; PBAs, primary bile acids; FXR, farnesoid X receptor; GUDCA, glycoursodeoxycholic acid; FMT, fecal microbiota transplantation; PPGR, postprandial glycemic response.

## References

[B1] Aoki R. Kamikado K. Suda W. Takii H. Mikami Y. Suganuma N.. (2017). A Proliferative Probiotic Bifidobacterium Strain in the Gut Ameliorates Progression of Metabolic Disorders *via* Microbiota Modulation and Acetate Elevation. Sci. Rep. 7, 43522. doi: 10.1038/srep43522 28252037 PMC5333160

[B2] Aron-Wisnewsky J. Clément K. Nieuwdorp M. (2019). Fecal Microbiota Transplantation: A Future Therapeutic Option for Obesity/Diabetes? Curr. Diabetes Rep. 19 (8), 51. doi: 10.1007/s11892-019-1180-z 31250122

[B3] Baggio L. L. Drucker D. J. (2014). Glucagon-Like Peptide-1 Receptors in the Brain: Controlling Food Intake and Body Weight. J. Clin. Invest. 124 (10), 4223–4226. doi: 10.1172/jci78371 25202976 PMC4191040

[B4] Bailey C. J. Wilcock C. Scarpello J. H. (2008). Metformin and the Intestine. Diabetologia 51 (8), 1552–1553. doi: 10.1007/s00125-008-1053-5 18528677

[B5] Bauer P. V. Duca F. A. Waise T. M. Z. Rasmussen B. A. Abraham M. A. Dranse H. J.. (2018). Metformin Alters Upper Small Intestinal Microbiota That Impact a Glucose-SGLT1-Sensing Glucoregulatory Pathway. Cell Metab. 27 (1), 101–117.e105. doi: 10.1016/j.cmet.2017.09.019 29056513

[B6] Birkeland E. Gharagozlian S. Birkeland K. I. Valeur J. Måge I. Rud I.. (2020). Prebiotic Effect of Inulin-Type Fructans on Faecal Microbiota and Short-Chain Fatty Acids in Type 2 Diabetes: A Randomised Controlled Trial. Eur. J. Nutr. 59 (7), 3325–3338. doi: 10.1007/s00394-020-02282-5 32440730 PMC7501097

[B7] Bock P. M. Telo G. H. Ramalho R. Sbaraini M. Leivas G. Martins A. F.. (2021). The Effect of Probiotics, Prebiotics or Synbiotics on Metabolic Outcomes in Individuals With Diabetes: A Systematic Review and Meta-Analysis. Diabetologia 64 (1), 26–41. doi: 10.1007/s00125-020-05295-1 33047170

[B8] Bonora E. Cigolini M. Bosello O. Zancanaro C. Capretti L. Zavaroni I.. (1984). Lack of Effect of Intravenous Metformin on Plasma Concentrations of Glucose, Insulin, C-Peptide, Glucagon and Growth Hormone in Non-Diabetic Subjects. Curr. Med. Res. Opin. 9 (1), 47–51. doi: 10.1185/03007998409109558 6373159

[B9] Bouter K. E. van Raalte D. H. Groen A. K. Nieuwdorp M. (2017). Role of the Gut Microbiome in the Pathogenesis of Obesity and Obesity-Related Metabolic Dysfunction. Gastroenterology 152 (7), 1671–1678. doi: 10.1053/j.gastro.2016.12.048 28192102

[B10] Bryrup T. Thomsen C. W. Kern T. Allin K. H. Brandslund I. Jørgensen N. R.. (2019). Metformin-Induced Changes of the Gut Microbiota in Healthy Young Men: Results of a non-Blinded, One-Armed Intervention Study. Diabetologia 62 (6), 1024–1035. doi: 10.1007/s00125-019-4848-7 30904939 PMC6509092

[B11] Cao T. T. B. Wu K. C. Hsu J. L. Chang C. S. Chou C. Lin C. Y.. (2020). Effects of Non-Insulin Anti-Hyperglycemic Agents on Gut Microbiota: A Systematic Review on Human and Animal Studies. Front. Endocrinol. (Lausanne) 11. doi: 10.3389/fendo.2020.573891 PMC7538596 33071980

[B12] Cao Y. Yao G. Sheng Y. Yang L. Wang Z. Yang Z.. (2019). JinQi Jiangtang Tablet Regulates Gut Microbiota and Improve Insulin Sensitivity in Type 2 Diabetes Mice. J. Diabetes Res. 2019, 1872134. doi: 10.1155/2019/1872134 30733971 PMC6348821

[B13] Cersosimo E.. (2000)"Pathogenesis of Type 2 Diabetes Mellitus,". In: Endotext (South Dartmouth (MA: MDText.com, Inc) (Accessed 12 Mar 2019).

[B14] Charpentier J. Briand F. Lelouvier B. Servant F. Azalbert V. Puel A.. (2021). Liraglutide Targets the Gut Microbiota and the Intestinal Immune System to Regulate Insulin Secretion. Acta Diabetol. 58 (7), 881–897. doi: 10.1007/s00592-020-01657-8 33723651

[B15] Chen M. Liao Z. Lu B. Wang M. Lin L. Zhang S.. (2018). Huang-Lian-Jie-Du-Decoction Ameliorates Hyperglycemia and Insulin Resistant in Association With Gut Microbiota Modulation. Front. Microbiol. 9. doi: 10.3389/fmicb.2018.02380 PMC6186778 30349514

[B16] Chen H. Yao Y. Wang W. Wang D. (2021). Ge-Gen-Jiao-Tai-Wan Affects Type 2 Diabetic Rats by Regulating Gut Microbiota and Primary Bile Acids. Evid. Based Complement. Alternat. Med. 2021, 5585952. doi: 10.1155/2021/5585952 33953783 PMC8064793

[B17] de la Cuesta-Zuluaga J. Mueller N. T. Corrales-Agudelo V. Velásquez-Mejía E. P. Carmona J. A. Abad J. M.. (2017). Metformin Is Associated With Higher Relative Abundance of Mucin-Degrading Akkermansia Muciniphila and Several Short-Chain Fatty Acid-Producing Microbiota in the Gut. Diabetes Care 40 (1), 54–62. doi: 10.2337/dc16-1324 27999002

[B18] Doestzada M. Vila A. V. Zhernakova A. Koonen D. P. Y. Weersma R. K. Touw D. J.. (2018). Pharmacomicrobiomics: A Novel Route Towards Personalized Medicine? Protein Cell 9 (5), 432–445. doi: 10.1007/s13238-018-0547-2 29705929 PMC5960471

[B19] Do H. J. Lee Y. S. Ha M. J. Cho Y. Yi H. Hwang Y. J.. (2016). Beneficial Effects of Voglibose Administration on Body Weight and Lipid Metabolism *via* Gastrointestinal Bile Acid Modification. Endocr. J. 63 (8), 691–702. doi: 10.1507/endocrj.EJ15-0747 27349182

[B20] Drucker D. J. Nauck M. A. (2006). The Incretin System: Glucagon-Like Peptide-1 Receptor Agonists and Dipeptidyl Peptidase-4 Inhibitors in Type 2 Diabetes. Lancet 368 (9548), 1696–1705. doi: 10.1016/s0140-6736(06)69705-5 17098089

[B21] Du F. Hinke S. A. Cavanaugh C. Polidori D. Wallace N. Kirchner T.. (2018). Potent Sodium/Glucose Cotransporter SGLT1/2 Dual Inhibition Improves Glycemic Control Without Marked Gastrointestinal Adaptation or Colonic Microbiota Changes in Rodents. J. Pharmacol. Exp. Ther. 365 (3), 676–687. doi: 10.1124/jpet.118.248575 29674332

[B22] Ejtahed H. S. Soroush A. R. Angoorani P. Larijani B. Hasani-Ranjbar S. (2016). Gut Microbiota as a Target in the Pathogenesis of Metabolic Disorders: A New Approach to Novel Therapeutic Agents. Horm. Metab. Res. 48 (6), 349–358. doi: 10.1055/s-0042-107792 27203411

[B23] Ejtahed H. S. Tito R. Y. Siadat S. D. Hasani-Ranjbar S. Hoseini-Tavassol Z. Rymenans L.. (2019). Metformin Induces Weight Loss Associated With Gut Microbiota Alteration in non-Diabetic Obese Women: A Randomized Double-Blind Clinical Trial. Eur. J. Endocrinol. 180 (3), 165–176. doi: 10.1530/eje-18-0826 30540558

[B24] Forslund K. Hildebrand F. Nielsen T. Falony G. Le Chatelier E. Sunagawa S.. (2015). Disentangling Type 2 Diabetes and Metformin Treatment Signatures in the Human Gut Microbiota. Nature 528 (7581), 262–266. doi: 10.1038/nature15766 26633628 PMC4681099

[B25] Ghorbani Y. Schwenger K. J. P. Allard J. P. (2021). Manipulation of Intestinal Microbiome as Potential Treatment for Insulin Resistance and Type 2 Diabetes. Eur. J. Nutr. 60 (5), 2361–2379. doi: 10.1007/s00394-021-02520-4 33651137

[B26] Gibson G. R. Roberfroid M. B. (1995). Dietary Modulation of the Human Colonic Microbiota: Introducing the Concept of Prebiotics. J. Nutr. 125 (6), 1401–1412. doi: 10.1093/jn/125.6.1401 7782892

[B27] Grasset E. Puel A. Charpentier J. Collet X. Christensen J. E. Tercé F.. (2017). A Specific Gut Microbiota Dysbiosis of Type 2 Diabetic Mice Induces GLP-1 Resistance Through an Enteric NO-Dependent and Gut-Brain Axis Mechanism. Cell Metab. 25 (5), 1075–1090.e1075. doi: 10.1016/j.cmet.2017.04.013 28467926

[B28] Groen A. K. Nieuwdorp M. (2017). An Evaluation of the Therapeutic Potential of Fecal Microbiota Transplantation to Treat Infectious and Metabolic Diseases. EMBO Mol. Med. 9 (1), 1–3. doi: 10.15252/emmm.201607035 27861129 PMC5210083

[B29] Gu Y. Wang X. Li J. Zhang Y. Zhong H. Liu R.. (2017). Analyses of Gut Microbiota and Plasma Bile Acids Enable Stratification of Patients for Antidiabetic Treatment. Nat. Commun. 8 (1), 1785. doi: 10.1038/s41467-017-01682-2 29176714 PMC5702614

[B30] Handelsman Y. Bloomgarden Z. T. Grunberger G. Umpierrez G. Zimmerman R. S. Bailey T. S.. (2015). American Association of Clinical Endocrinologists and American College of Endocrinology - Clinical Practice Guidelines for Developing a Diabetes Mellitus Comprehensive Care Plan - 2015. Endocr. Pract. 21 Suppl 1 (Suppl 1), 1–87. doi: 10.4158/ep15672.gl 25869408 PMC4959114

[B31] Heinken A. Basile A. Hertel J. Thinnes C. Thiele I. (2021). Genome-Scale Metabolic Modeling of the Human Microbiome in the Era of Personalized Medicine. Annu. Rev. Microbiol. 75, 199–222. doi: 10.1146/annurev-micro-060221-012134 34314593

[B32] He C. Shan Y. Song W. (2015). Targeting Gut Microbiota as a Possible Therapy for Diabetes. Nutr. Res. 35 (5), 361–367. doi: 10.1016/j.nutres.2015.03.002 25818484

[B33] Hill C. Guarner F. Reid G. Gibson G. R. Merenstein D. J. Pot B.. (2014). Expert Consensus Document. The International Scientific Association for Probiotics and Prebiotics Consensus Statement on the Scope and Appropriate Use of the Term Probiotic. Nat. Rev. Gastroenterol. Hepatol. 11 (8), 506–514. doi: 10.1038/nrgastro.2014.66 24912386

[B34] Hsieh P. S. Ho H. H. Tsao S. P. Hsieh S. H. Lin W. Y. Chen J. F.. (2021). Multi-Strain Probiotic Supplement Attenuates Streptozotocin-Induced Type-2 Diabetes by Reducing Inflammation and β-Cell Death in Rats. PloS One 16 (6), e0251646. doi: 10.1371/journal.pone.0251646 34166387 PMC8224959

[B35] Huda M. N. Kim M. Bennett B. J. (2021). Modulating the Microbiota as a Therapeutic Intervention for Type 2 Diabetes. Front. Endocrinol. (Lausanne) 12. doi: 10.3389/fendo.2021.632335 PMC8060771 33897618

[B36] Huo T. Xiong Z. Lu X. Cai S. (2015). Metabonomic Study of Biochemical Changes in Urinary of Type 2 Diabetes Mellitus Patients After the Treatment of Sulfonylurea Antidiabetic Drugs Based on Ultra-Performance Liquid Chromatography/Mass Spectrometry. BioMed. Chromatogr. 29 (1), 115–122. doi: 10.1002/bmc.3247 24890121

[B37] Hu Y. Zhou X. Liu P. Wang B. Duan D. M. Guo D. H. (2016). A Comparison Study of Metformin Only Therapy and Metformin Combined With Chinese Medicine Jianyutangkang Therapy in Patients With Type 2 Diabetes: A Randomized Placebo-Controlled Double-Blind Study. Complement. Ther. Med. 24, 13–18. doi: 10.1016/j.ctim.2015.11.005 26860796

[B38] Javdan B. Lopez J. G. Chankhamjon P. Lee Y. J. Hull R. Wu Q.. (2020). Personalized Mapping of Drug Metabolism by the Human Gut Microbiome. Cell 181 (7), 1661–1679.e1622. doi: 10.1016/j.cell.2020.05.001 32526207 PMC8591631

[B39] Kesika P. Sivamaruthi B. S. Chaiyasut C. (2019). Do Probiotics Improve the Health Status of Individuals With Diabetes Mellitus? A Review on Outcomes of Clinical Trials. BioMed. Res. Int. 2019, 1531567. doi: 10.1155/2019/1531567 31950031 PMC6949658

[B40] Khoruts A. Sadowsky M. J. (2016). Understanding the Mechanisms of Faecal Microbiota Transplantation. Nat. Rev. Gastroenterol. Hepatol. 13 (9), 508–516. doi: 10.1038/nrgastro.2016.98 27329806 PMC5909819

[B41] Kim Y. A. Keogh J. B. Clifton P. M. (2018). Probiotics, Prebiotics, Synbiotics and Insulin Sensitivity. Nutr. Res. Rev. 31 (1), 35–51. doi: 10.1017/s095442241700018x 29037268

[B42] Kimura I. Ozawa K. Inoue D. Imamura T. Kimura K. Maeda T.. (2013). The Gut Microbiota Suppresses Insulin-Mediated Fat Accumulation *via* the Short-Chain Fatty Acid Receptor GPR43. Nat. Commun. 4, 1829. doi: 10.1038/ncomms2852 23652017 PMC3674247

[B43] Knop F. K. Aaboe K. Vilsbøll T. Vølund A. Holst J. J. Krarup T.. (2012). Impaired Incretin Effect and Fasting Hyperglucagonaemia Characterizing Type 2 Diabetic Subjects are Early Signs of Dysmetabolism in Obesity. Diabetes Obes. Metab. 14 (6), 500–510. doi: 10.1111/j.1463-1326.2011.01549.x 22171657

[B44] Kootte R. S. Levin E. Salojärvi J. Smits L. P. Hartstra A. V. Udayappan S. D.. (2017). Improvement of Insulin Sensitivity After Lean Donor Feces in Metabolic Syndrome Is Driven by Baseline Intestinal Microbiota Composition. Cell Metab. 26 (4), 611–619.e616. doi: 10.1016/j.cmet.2017.09.008 28978426

[B45] Koppel N. Maini Rekdal V. Balskus E. P. (2017). Chemical Transformation of Xenobiotics by the Human Gut Microbiota. Science 356 (6344), eaag2770. doi: 10.1126/science.aag2770 28642381 PMC5534341

[B46] Koropatkin N. M. Martens E. C. (2017). Meds Modify Microbiome, Mediating Their Effects. Cell Metab. 26 (3), 456–457. doi: 10.1016/j.cmet.2017.08.022 28877452

[B47] Korpela K. Flint H. J. Johnstone A. M. Lappi J. Poutanen K. Dewulf E.. (2014). Gut Microbiota Signatures Predict Host and Microbiota Responses to Dietary Interventions in Obese Individuals. PloS One 9 (6), e90702. doi: 10.1371/journal.pone.0090702 24603757 PMC3946202

[B48] Kovatcheva-Datchary P. Nilsson A. Akrami R. Lee Y. S. De Vadder F. Arora T.. (2015). Dietary Fiber-Induced Improvement in Glucose Metabolism Is Associated With Increased Abundance of Prevotella. Cell Metab. 22 (6), 971–982. doi: 10.1016/j.cmet.2015.10.001 26552345

[B49] Kuriyama C. Kamiyama O. Ikeda K. Sanae F. Kato A. Adachi I.. (2008). *In Vitro* Inhibition of Glycogen-Degrading Enzymes and Glycosidases by Six-Membered Sugar Mimics and Their Evaluation in Cell Cultures. Bioorg. Med. Chem. 16 (15), 7330–7336. doi: 10.1016/j.bmc.2008.06.026 18595718

[B50] Lagier J. C. Khelaifia S. Alou M. T. Ndongo S. Dione N. Hugon P.. (2016). Culture of Previously Uncultured Members of the Human Gut Microbiota by Culturomics. Nat. Microbiol. 1, 16203. doi: 10.1038/nmicrobiol.2016.203 27819657 PMC12094094

[B51] Lampe J. W. Navarro S. L. Hullar M. A. Shojaie A. (2013). Inter-Individual Differences in Response to Dietary Intervention: Integrating Omics Platforms Towards Personalised Dietary Recommendations. Proc. Nutr. Soc. 72 (2), 207–218. doi: 10.1017/s0029665113000025 23388096 PMC3694579

[B52] Lee D. M. Battson M. L. Jarrell D. K. Hou S. Ecton K. E. Weir T. L.. (2018). SGLT2 Inhibition *via* Dapagliflozin Improves Generalized Vascular Dysfunction and Alters the Gut Microbiota in Type 2 Diabetic Mice. Cardiovasc. Diabetol. 17 (1), 62. doi: 10.1186/s12933-018-0708-x 29703207 PMC5921754

[B53] Lee H. Ko G. (2014). Effect of Metformin on Metabolic Improvement and Gut Microbiota. Appl. Environ. Microbiol. 80 (19), 5935–5943. doi: 10.1128/aem.01357-14 25038099 PMC4178684

[B54] Lee Y. S. Lee D. Park G. S. Ko S. H. Park J. Lee Y. K.. (2021). Lactobacillus Plantarum HAC01 Ameliorates Type 2 Diabetes in High-Fat Diet and Streptozotocin-Induced Diabetic Mice in Association With Modulating the Gut Microbiota. Food Funct. 12 (14), 6363–6373. doi: 10.1039/d1fo00698c 34105563

[B55] Lian F. Tian J. Chen X. Li Z. Piao C. Guo J.. (2015). The Efficacy and Safety of Chinese Herbal Medicine Jinlida as Add-On Medication in Type 2 Diabetes Patients Ineffectively Managed by Metformin Monotherapy: A Double-Blind, Randomized, Placebo-Controlled, Multicenter Trial. PloS One 10 (6), e0130550. doi: 10.1371/journal.pone.0130550 26098833 PMC4476735

[B56] Liao X. Song L. Zeng B. Liu B. Qiu Y. Qu H.. (2019). Alteration of Gut Microbiota Induced by DPP-4i Treatment Improves Glucose Homeostasis. EBioMedicine 44, 665–674. doi: 10.1016/j.ebiom.2019.03.057 30922964 PMC6603491

[B57] Li K. Zhang L. Xue J. Yang X. Dong X. Sha L.. (2019). Dietary Inulin Alleviates Diverse Stages of Type 2 Diabetes Mellitus *via* Anti-Inflammation and Modulating Gut Microbiota in Db/Db Mice. Food Funct. 10 (4), 1915–1927. doi: 10.1039/c8fo02265h 30869673

[B58] Madsen M. S. A. Grønlund R. V. Eid J. Christensen-Dalsgaard M. Sommer M. Rigbolt K.. (2021). Characterization of Local Gut Microbiome and Intestinal Transcriptome Responses to Rosiglitazone Treatment in Diabetic Db/Db Mice. BioMed. Pharmacother. 133, 110966. doi: 10.1016/j.biopha.2020.110966 33171401

[B59] Mahboobi S. Rahimi F. Jafarnejad S. (2018). Effects of Prebiotic and Synbiotic Supplementation on Glycaemia and Lipid Profile in Type 2 Diabetes: A Meta-Analysis of Randomized Controlled Trials. Adv. Pharm. Bull. 8 (4), 565–574. doi: 10.15171/apb.2018.065 30607329 PMC6311648

[B60] Montandon S. A. Jornayvaz F. R. (2017). Effects of Antidiabetic Drugs on Gut Microbiota Composition. Genes (Basel) 8 (10), 250. doi: 10.3390/genes8100250 PMC5664100 28973971

[B61] Morshedi M. Saghafi-Asl M. Hosseinifard E. S. (2020). The Potential Therapeutic Effects of the Gut Microbiome Manipulation by Synbiotic Containing-Lactobacillus Plantarum on Neuropsychological Performance of Diabetic Rats. J. Transl. Med. 18 (1), 18. doi: 10.1186/s12967-019-02169-y 31924200 PMC6953298

[B62] Mueller N. T. Differding M. K. Zhang M. Maruthur N. M. Juraschek S. P. Miller E. R. 3rd. (2021). Metformin Affects Gut Microbiome Composition and Function and Circulating Short-Chain Fatty Acids: A Randomized Trial. Diabetes Care 44 (7), 1462–1471. doi: 10.2337/dc20-2257 34006565 PMC8323185

[B63] Mulder T. P. Rietveld A. G. van Amelsvoort J. M. (2005). Consumption of Both Black Tea and Green Tea Results in an Increase in the Excretion of Hippuric Acid Into Urine. Am. J. Clin. Nutr. 81 (1 Suppl), 256s–260s. doi: 10.1093/ajcn/81.1.256S 15640488

[B64] Nakajima H. Takewaki F. Hashimoto Y. Kajiyama S. Majima S. Okada H.. (2020). The Effects of Metformin on the Gut Microbiota of Patients With Type 2 Diabetes: A Two-Center, Quasi-Experimental Study. Life (Basel) 10 (9), 195. doi: 10.3390/life10090195 PMC7555986 32932871

[B65] Natori Y. Imahori T. Murakami K. Yoshimura Y. Nakagawa S. Kato A.. (2011). The Synthesis and Biological Evaluation of 1-C-Alkyl-L-Arabinoiminofuranoses, a Novel Class of α-Glucosidase Inhibitors. Bioorg. Med. Chem. Lett. 21 (2), 738–741. doi: 10.1016/j.bmcl.2010.11.112 21185187

[B66] Nie Q. Chen H. Hu J. Fan S. Nie S. (2019). Dietary Compounds and Traditional Chinese Medicine Ameliorate Type 2 Diabetes by Modulating Gut Microbiota. Crit. Rev. Food Sci. Nutr. 59 (6), 848–863. doi: 10.1080/10408398.2018.1536646 30569745

[B67] Olivares M. Neyrinck A. M. Pötgens S. A. Beaumont M. Salazar N. Cani P. D.. (2018a). The DPP-4 Inhibitor Vildagliptin Impacts the Gut Microbiota and Prevents Disruption of Intestinal Homeostasis Induced by a Western Diet in Mice. Diabetologia 61 (8), 1838–1848. doi: 10.1007/s00125-018-4647-6 29797022 PMC6061172

[B68] Olivares M. Schüppel V. Hassan A. M. Beaumont M. Neyrinck A. M. Bindels L. B.. (2018b). The Potential Role of the Dipeptidyl Peptidase-4-Like Activity From the Gut Microbiota on the Host Health. Front. Microbiol. 9, 1900. doi: 10.3389/fmicb.2018.01900 30186247 PMC6113382

[B69] Palacios T. Vitetta L. Coulson S. Madigan C. D. Lam Y. Y. Manuel R.. (2020). Targeting the Intestinal Microbiota to Prevent Type 2 Diabetes and Enhance the Effect of Metformin on Glycaemia: A Randomised Controlled Pilot Study. Nutrients 12 (7), 2041. doi: 10.3390/nu12072041 PMC7400852 32660025

[B70] Salonen A. Lahti L. Salojärvi J. Holtrop G. Korpela K. Duncan S. H.. (2014). Impact of Diet and Individual Variation on Intestinal Microbiota Composition and Fermentation Products in Obese Men. Isme J. 8 (11), 2218–2230. doi: 10.1038/ismej.2014.63 24763370 PMC4992075

[B71] Shang J. Liu F. Zhang B. Dong K. Lu M. Jiang R.. (2021). Liraglutide-Induced Structural Modulation of the Gut Microbiota in Patients With Type 2 Diabetes Mellitus. PeerJ 9, e11128. doi: 10.7717/peerj.11128 33850659 PMC8019531

[B72] Sharma P. Bhardwaj P. Singh R. (2016). Administration of Lactobacillus Casei and Bifidobacterium Bifidum Ameliorated Hyperglycemia, Dyslipidemia, and Oxidative Stress in Diabetic Rats. Int. J. Prev. Med. 7, 102. doi: 10.4103/2008-7802.188870 27625767 PMC5007903

[B73] Shilo S. Godneva A. Rachmiel M. Korem T. Kolobkov D. Karady T.. (2022). Prediction of Personal Glycemic Responses to Food for Individuals With Type 1 Diabetes Through Integration of Clinical and Microbial Data. Diabetes Care 45 (3), 502–511. doi: 10.2337/dc21-1048 34711639

[B74] Smith B. J. Miller R. A. Ericsson A. C. Harrison D. C. Strong R. Schmidt T. M. (2019). Changes in the Gut Microbiome and Fermentation Products Concurrent With Enhanced Longevity in Acarbose-Treated Mice. BMC Microbiol. 19 (1), 130. doi: 10.1186/s12866-019-1494-7 31195972 PMC6567620

[B75] Spanogiannopoulos P. Bess E. N. Carmody R. N. Turnbaugh P. J. (2016). The Microbial Pharmacists Within Us: A Metagenomic View of Xenobiotic Metabolism. Nat. Rev. Microbiol. 14 (5), 273–287. doi: 10.1038/nrmicro.2016.17 26972811 PMC5243131

[B76] Su B. Liu H. Li J. Sunli Y. Liu B. Liu D.. (2015). Acarbose Treatment Affects the Serum Levels of Inflammatory Cytokines and the Gut Content of Bifidobacteria in Chinese Patients With Type 2 Diabetes Mellitus. J. Diabetes 7 (5), 729–739. doi: 10.1111/1753-0407.12232 25327485

[B77] Su H. Mo J. Ni J. Ke H. Bao T. Xie J.. (2020). Andrographolide Exerts Antihyperglycemic Effect Through Strengthening Intestinal Barrier Function and Increasing Microbial Composition of Akkermansia Muciniphila. Oxid. Med. Cell Longev. 2020, 6538930. doi: 10.1155/2020/6538930 32774682 PMC7396114

[B78] Sun L. Xie C. Wang G. Wu Y. Wu Q. Wang X.. (2018). Gut Microbiota and Intestinal FXR Mediate the Clinical Benefits of Metformin. Nat. Med. 24 (12), 1919–1929. doi: 10.1038/s41591-018-0222-4 30397356 PMC6479226

[B79] Tahrani A. A. Barnett A. H. Bailey C. J. (2013). SGLT Inhibitors in Management of Diabetes. Lancet Diabetes Endocrinol. 1 (2), 140–151. doi: 10.1016/s2213-8587(13)70050-0 24622320

[B80] Tan K. Tesar C. Wilton R. Jedrzejczak R. P. Joachimiak A. (2018). Interaction of Antidiabetic α-Glucosidase Inhibitors and Gut Bacteria α-Glucosidase. Protein Sci. 27 (8), 1498–1508. doi: 10.1002/pro.3444 29761590 PMC6153411

[B81] Toejing P. Khampithum N. Sirilun S. Chaiyasut C. Lailerd N. (2021). Influence of Lactobacillus Paracasei HII01 Supplementation on Glycemia and Inflammatory Biomarkers in Type 2 Diabetes: A Randomized Clinical Trial. Foods 10 (7), 1455. doi: 10.3390/foods10071455 34201653 PMC8303256

[B82] Tolhurst G. Heffron H. Lam Y. S. Parker H. E. Habib A. M. Diakogiannaki E.. (2012). Short-Chain Fatty Acids Stimulate Glucagon-Like Peptide-1 Secretion *via* the G-Protein-Coupled Receptor FFAR2. Diabetes 61 (2), 364–371. doi: 10.2337/db11-1019 22190648 PMC3266401

[B83] van Bommel E. J. M. Herrema H. Davids M. Kramer M. H. H. Nieuwdorp M. van Raalte D. H. (2020). Effects of 12-Week Treatment With Dapagliflozin and Gliclazide on Faecal Microbiome: Results of a Double-Blind Randomized Trial in Patients With Type 2 Diabetes. Diabetes Metab. 46 (2), 164–168. doi: 10.1016/j.diabet.2019.11.005 31816432

[B84] Vázquez-Baeza Y. Callewaert C. Debelius J. Hyde E. Marotz C. Morton J. T.. (2018). Impacts of the Human Gut Microbiome on Therapeutics. Annu. Rev. Pharmacol. Toxicol. 58, 253–270. doi: 10.1146/annurev-pharmtox-042017-031849 28968189

[B85] Vettorazzi J. F. Ribeiro R. A. Borck P. C. Branco R. C. Soriano S. Merino B.. (2016). The Bile Acid TUDCA Increases Glucose-Induced Insulin Secretion *via* the cAMP/PKA Pathway in Pancreatic Beta Cells. Metabolism 65 (3), 54–63. doi: 10.1016/j.metabol.2015.10.021 26892516

[B86] Wang L. Li P. Tang Z. Yan X. Feng B. (2016). Structural Modulation of the Gut Microbiota and the Relationship With Body Weight: Compared Evaluation of Liraglutide and Saxagliptin Treatment. Sci. Rep. 6, 33251. doi: 10.1038/srep33251 27633081 PMC5025740

[B87] Wang Y. Tong Q. Shou J. W. Zhao Z. X. Li X. Y. Zhang X. F.. (2017). Gut Microbiota-Mediated Personalized Treatment of Hyperlipidemia Using Berberine. Theranostics 7 (9), 2443–2451. doi: 10.7150/thno.18290 28744326 PMC5525748

[B88] Wang Y. Wu Y. Sailike J. Sun X. Abuduwaili N. Tuoliuhan H.. (2020). Fourteen Composite Probiotics Alleviate Type 2 Diabetes Through Modulating Gut Microbiota and Modifying M1/M2 Phenotype Macrophage in Db/Db Mice. Pharmacol. Res. 161, 105150. doi: 10.1016/j.phrs.2020.105150 32818655

[B89] Wei X. Tao J. Xiao S. Jiang S. Shang E. Zhu Z.. (2018). Xiexin Tang Improves the Symptom of Type 2 Diabetic Rats by Modulation of the Gut Microbiota. Sci. Rep. 8 (1), 3685. doi: 10.1038/s41598-018-22094-2 29487347 PMC5829262

[B90] Williams R. E. Eyton-Jones H. W. Farnworth M. J. Gallagher R. Provan W. M. (2002). Effect of Intestinal Microflora on the Urinary Metabolic Profile of Rats: A (1)H-Nuclear Magnetic Resonance Spectroscopy Study. Xenobiotica 32 (9), 783–794. doi: 10.1080/00498250210143047 12396275

[B91] Woting A. Blaut M. (2016). The Intestinal Microbiota in Metabolic Disease. Nutrients 8 (4), 202. doi: 10.3390/nu8040202 27058556 PMC4848671

[B92] Wu G. D. Chen J. Hoffmann C. Bittinger K. Chen Y. Y. Keilbaugh S. A.. (2011). Linking Long-Term Dietary Patterns With Gut Microbial Enterotypes. Science 334 (6052), 105–108. doi: 10.1126/science.1208344 21885731 PMC3368382

[B93] Wu H. Esteve E. Tremaroli V. Khan M. T. Caesar R. Mannerås-Holm L.. (2017). Metformin Alters the Gut Microbiome of Individuals With Treatment-Naive Type 2 Diabetes, Contributing to the Therapeutic Effects of the Drug. Nat. Med. 23 (7), 850–858. doi: 10.1038/nm.4345 28530702

[B94] Xu X. Gao Z. Yang F. Yang Y. Chen L. Han L.. (2020). Antidiabetic Effects of Gegen Qinlian Decoction *via* the Gut Microbiota Are Attributable to Its Key Ingredient Berberine. Genomics Proteomics Bioinf. 18 (6), 721–736 doi: 10.1016/j.gpb.2019.09.007 PMC8377040 33359679

[B95] Xu T. Ge Y. Du H. Li Q. Xu X. Yi H.. (2021). Berberis Kansuensis Extract Alleviates Type 2 Diabetes in Rats by Regulating Gut Microbiota Composition. J. Ethnopharmacol. 273, 113995. doi: 10.1016/j.jep.2021.113995 33675912

[B96] Xu J. Lian F. Zhao L. Zhao Y. Chen X. Zhang X.. (2015). Structural Modulation of Gut Microbiota During Alleviation of Type 2 Diabetes With a Chinese Herbal Formula. Isme J. 9 (3), 552–562. doi: 10.1038/ismej.2014.177 25279787 PMC4331591

[B97] Yan X. Feng B. Li P. Tang Z. Wang L. (2016). Microflora Disturbance During Progression of Glucose Intolerance and Effect of Sitagliptin: An Animal Study. J. Diabetes Res. 2016, 2093171. doi: 10.1155/2016/2093171 27631013 PMC5007364

[B98] Yang M. Shi F. H. Liu W. Zhang M. C. Feng R. L. Qian C.. (2020). Dapagliflozin Modulates the Fecal Microbiota in a Type 2 Diabetic Rat Model. Front. Endocrinol. (Lausanne) 11. doi: 10.3389/fendo.2020.00635 PMC7707060 33312157

[B99] Yao Y. Chen H. Yan L. Wang W. Wang D. (2020). Berberine Alleviates Type 2 Diabetic Symptoms by Altering Gut Microbiota and Reducing Aromatic Amino Acids. BioMed. Pharmacother. 131, 110669. doi: 10.1016/j.biopha.2020.110669 32937246

[B100] Yuan X. Ni H. Chen X. Feng X. Wu Q. Chen J. (2018). Identification of Therapeutic Effect of Glucagon-Like Peptide 1 in the Treatment of STZ-Induced Diabetes Mellitus in Rats by Restoring the Balance of Intestinal Flora. J. Cell Biochem. 119 (12), 10067–10074. doi: 10.1002/jcb.27343 30129059

[B101] Yu L. Zhou X. Duan H. Chen Y. Cui S. Guo R.. (2021). Synergistic Protective Effects of Different Dietary Supplements Against Type 2 Diabetes *via* Regulating Gut Microbiota. J. Med. Food 24 (3), 319–330. doi: 10.1089/jmf.2020.4759 33739885

[B102] Zhang X. Fang Z. Zhang C. Xia H. Jie Z. Han X.. (2017). Effects of Acarbose on the Gut Microbiota of Prediabetic Patients: A Randomized, Double-Blind, Controlled Crossover Trial. Diabetes Ther. 8 (2), 293–307. doi: 10.1007/s13300-017-0226-y 28130771 PMC5380489

[B103] Zhang Y. Gu Y. Ren H. Wang S. Zhong H. Zhao X.. (2020). Gut Microbiome-Related Effects of Berberine and Probiotics on Type 2 Diabetes (the PREMOTE Study). Nat. Commun. 11 (1), 5015. doi: 10.1038/s41467-020-18414-8 33024120 PMC7538905

[B104] Zhang P. P. Li L. L. Han X. Li Q. W. Zhang X. H. Liu J. J.. (2020). Fecal Microbiota Transplantation Improves Metabolism and Gut Microbiome Composition in Db/Db Mice. Acta Pharmacol. Sin. 41 (5), 678–685. doi: 10.1038/s41401-019-0330-9 31937933 PMC7468362

[B105] Zhang F. Wang M. Yang J. Xu Q. Liang C. Chen B.. (2019). Response of Gut Microbiota in Type 2 Diabetes to Hypoglycemic Agents. Endocrine 66 (3), 485–493. doi: 10.1007/s12020-019-02041-5 31410749

[B106] Zhang Q. Xiao X. Li M. Yu M. Ping F. Zheng J.. (2017). Vildagliptin Increases Butyrate-Producing Bacteria in the Gut of Diabetic Rats. PloS One 12 (10), e0184735. doi: 10.1371/journal.pone.0184735 29036231 PMC5643055

[B107] Zhang Q. Xiao X. Zheng J. Li M. Yu M. Ping F.. (2018). Featured Article: Structure Moderation of Gut Microbiota in Liraglutide-Treated Diabetic Male Rats. Exp. Biol. Med. (Maywood) 243 (1), 34–44. doi: 10.1177/1535370217743765 29171288 PMC5788162

[B108] Zhang B. Yue R. Chen Y. Yang M. Huang X. Shui J.. (2019). Gut Microbiota, a Potential New Target for Chinese Herbal Medicines in Treating Diabetes Mellitus. Evid. Based Complement. Alternat. Med. 2019, 2634898. doi: 10.1155/2019/2634898 30906411 PMC6398116

[B109] Zhao L. Chen Y. Xia F. Abudukerimu B. Zhang W. Guo Y.. (2018). A Glucagon-Like Peptide-1 Receptor Agonist Lowers Weight by Modulating the Structure of Gut Microbiota. Front. Endocrinol. (Lausanne) 9. doi: 10.3389/fendo.2018.00233 PMC5966539 29867765

[B110] Zheng Y. Ding Q. Zhang L. Gou X. Wei Y. Li M.. (2020a). The Effect of Traditional Chinese Medicine on Gut Microbiota in Adults With Type 2 Diabetes: A Protocol for Systematic Review and Meta-Analysis. Med. (Baltimore) 99 (38), e22233. doi: 10.1097/md.0000000000022233 PMC7505378 32957365

[B111] Zheng Y. Gou X. Zhang L. Gao H. Wei Y. Yu X.. (2020b). Interactions Between Gut Microbiota, Host, and Herbal Medicines: A Review of New Insights Into the Pathogenesis and Treatment of Type 2 Diabetes. Front. Cell Infect. Microbiol. 10. doi: 10.3389/fcimb.2020.00360 PMC7379170 32766169

[B112] Zimmermann M. Zimmermann-Kogadeeva M. Wegmann R. Goodman A. L. (2019). Mapping Human Microbiome Drug Metabolism by Gut Bacteria and Their Genes. Nature 570 (7762), 462–467. doi: 10.1038/s41586-019-1291-3 31158845 PMC6597290

